# *Staphylococcus aureus* Interaction with Phospholipid Vesicles – A New Method to Accurately Determine Accessory Gene Regulator (*agr*) Activity

**DOI:** 10.1371/journal.pone.0087270

**Published:** 2014-01-30

**Authors:** Maisem Laabei, W. David Jamieson, Ruth C. Massey, A. Tobias A. Jenkins

**Affiliations:** 1 Department of Chemistry, University of Bath, Claverton Down, Bath, United Kingdom; 2 Department of Biology and Biochemistry, University of Bath, Claverton Down, Bath, United Kingdom; Universitätsklinikum Hamburg-Eppendorf, Germany

## Abstract

The staphylococcal accessory gene regulatory (*agr*) operon is a well-characterised global regulatory element that is important in the control of virulence gene expression for *Staphylococcus aureus*, a major human pathogen. Hence, accurate and sensitive measurement of Agr activity is central in understanding the virulence potential of *Staphylococcus aureus*, especially in the context of Agr dysfunction, which has been linked with persistent bacteraemia and reduced susceptibility to glycopeptide antibiotics. Agr function is typically measured using a synergistic haemolysis CAMP assay, which is believe to report on the level of expression of one of the translated products of the *agr* locus, delta toxin. In this study we develop a vesicle lysis test (VLT) that is specific to small amphipathic peptides, most notably delta and Phenol Soluble Modulin (PSM) toxins. To determine the accuracy of this VLT method in assaying Agr activity, we compared it to the CAMP assay using 89 clinical *Staphylococcus aureus* isolates. Of the 89 isolates, 16 were designated as having dysfunctional Agr systems by the CAMP assay, whereas only three were designated as such by VLT. Molecular analysis demonstrated that of these 16 isolates, the 13 designated as having a functional Agr system by VLT transcribed *rnaIII* and secreted delta toxin, demonstrating they have a functional Agr system despite the results of the CAMP assay. The *agr* locus of all 16 isolates was sequenced, and only the 3 designated as having a dysfunctional Agr system contained mutations, explaining their Agr dysfunction. Given the potentially important link between Agr dysfunction and clinical outcome, we have developed an assay that determines this more accurately than the conventional CAMP assay.

## Introduction

*Staphylococcus aureus* is a Gram-positive bacterial pathogen that plays a major role in human disease. It is an opportunistic pathogen which inhabits the moist squamous epithelium of the anterior nares, permanently colonising approximately 20% of the population and found intermittently in another 50% [1]. This organism can cause a wide array of diseases from superficial skin lesions to more serious life-threatening illnesses, being the leading bacterial agent in infective endocarditis [2], [3], [4]. *S. aureus* expresses many different secreted and surface-associated virulence factors which are utilised during all stages of infection [5].

The staphylococcal accessory gene regulator (*agr*) quorum sensing system is central in the ability of this organism to promote infection, through coordinated, temporal expression of specific virulence genes, in which cell surface adhesins are synthesised and expressed before secreted toxins and enzymes [6], [7]. Previous studies have shown that a functional Agr system is important in several infection models including a murine arthritic [8] and subcutaneous abscesses model [9] as well as rabbit endocarditis [10]. The shift in gene expression is tightly correlated with population density and sensing of a diffusible signal molecule by the Agr two-component system. The Agr system is comprised of a 3 kb locus with two divergent transcripts, RNAII and RNAIII, driven by independent promoters P2 and P3. The P2 operon encodes the two-component system, AgrA and C, which responds to the autoinducing peptide AgrD, after processing and secretion through AgrB, and after reaching a critical peptide threshold concentration, allowing a phosphorylated form of AgrA to drive expression from the two *agr* promoters. The P3 dependent regulatory RNA, RNAIII, is the effector molecule of this regulon and is involved in the up - and down - regulation of specific genes as well as encoding the delta toxin. This complex alteration in expression of virulence genes occurs in conjunction with many global regulators namely the DNA-binding Sar family of proteins [11], [12], the alternative sigma factor [13] and other two-component system such as SaeRS [14] and ArlRS [15].

Recently, there has been much research into Agr dysfunction, particularly on the outcomes of infection with isolates containing mutations in the *agr* locus, which can range in prevalence from 10–20% of *S. aureus* clinical isolates [16], [17]. These mutations can occur during infection in the patient [18] and have been implicated in an increased mortality in patients suffering from bacteremia [17], [19]. The increased survival of Agr defective strains observed in persistent bacteremia has been hypothesised to involve a defect in autolysis [20], owing to the fact that several murein-hydrolase genes are regulated by *agr* [21]. This phenotype has been implicated in the increased survival of Agr dysfunctional strains in the presence of platelet derived antimicrobial peptides and reduced sensitivity to gylcopeptide antibiotics, namely vancomycin [20].

The conventional method of assessing Agr function is to demonstrate delta toxin’s haemolytic activity using a blood agar plate assay in combination with a beta haemolysin positive strain, usually RN4220 [22]. Additionally, Agr activity can be monitored through other methods such as Northern blotting or quantitative reverse transcriptase-PCR (qRT-qPCR) with probes or primers directed at RNA III, and recently Whole-Cell Matrix Assisted Laser Desorption Ionization-Time-of-Flight (MALDI-TOF) mass spectrometry [23]. However, Northern blotting and qRT-PCR are time consuming and expensive, but considerably more sensitive than the CAMP assay, and a MALDI-TOF mass spectrometer costs several hundred thousand dollars to purchase, although running costs are low [24]. Here we describe the development of a new methodology that determines Agr activity in a fast, high-throughput, sensitive and quantitative manner. This method is based on the interaction of delta and Phenol Soluble Modulin (PSM) toxins, both used as surrogate markers for RNAIII and RNAII activity respectively, with lipid vesicles containing encapsulated self-quenched fluorescent dye. Recent work in this group has focused on the development of a sensor for bacterial infection in burns [25], [26] which is dependent on toxin-lipid interaction. One fundamental element in the transition from inactive toxin monomers into fully functional membrane-damaging agents is the lipid and protein composition of the target membranes, particularly important in artificial membranes. We have formulated a lipid vesicle system that is responsive to specific toxins, the small α-helical, amphiphatic delta and PSM toxins. When compared to the conventional CAMP assay this method proved more accurate, identifying all the isolates in a collection of 89 clinical *S. aureus* isolates that had mutations in the *agr* locus, unlike the CAMP assay that had 13 false positives. As such, if Agr dysfunction becomes more widely accepted as being a critical determinant in the clinical outcome of infection, the accuracy of the VLT assay suggests that it should be used ahead of the plate based CAMP assay.

## Results and Discussion

### Vesicle Breakdown Correlates with Early Stationary Phase Growth and is *agr* Dependent

**Figure 1:**
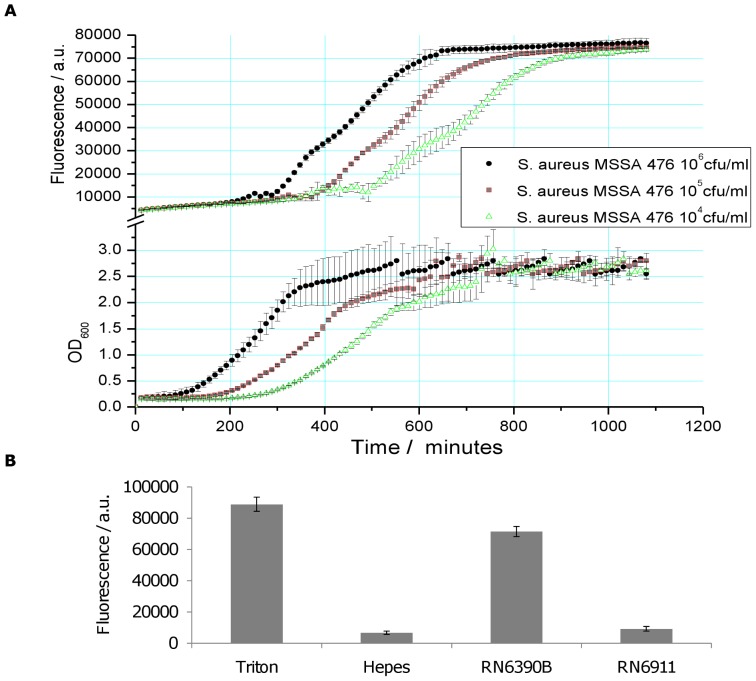
Vesicle lysis occurs during early stationary phase growth. **A)** Different starting inocula (10^4^, 10^5^, and 10^6 ^cfu/ml) of MSSA 476 were used to assess vesicle lysis during bacterial growth. By monitoring optical density and fluorescence it was evident that vesicle lysis occurs at early stationary phase. Bacteria were grown for 18 h with lipid vesicles **B)** Supernatants of wild-type (RN6390B) and the isogenic *agr* knockout (RN6911) strains, highlights the central role that the *agr* operon plays in vesicle lysis. 0.01% Triton X-100 and HEPES buffer were used as positive and negative controls respectively Supernatants were incubated with lipid vesicles for 30 min and fluorescence measured every 2 min.

Methicillin-susceptible *S. aureus* strain MSSA476 is a community-acquired invasive isolate for which a genome sequence is available [27]. To determine if and at what stage of growth MSSA476 lysed the lipid vesicles, a range of concentrations of bacteria were used to inoculate broth containing the vesicles. Toxin production in bacteria is strictly growth-phase regulated and density dependent, with bacteria relying on the secretion of auto-inducing peptides to communicate and regulate genes [28]. Figure 1(a) (top) shows the vesicle breakdown/fluorescence response, observed for the three start inoculums to be growth phase dependent; being triggered in the early stationary phase in each case. Due to the requirement of bacteria reaching stationary phase, the role of quorum sensing and the *agr* operon was investigated. Supernatants derived from wild type RN6390B and isogenic *agr* knockout RN6911 were used (fig. 1b) to measure the fluorescence response of vesicles to secreted toxins. It was observed that the wild type but not the isogenic *agr* mutant strain caused lysis, demonstrating the importance of a functional *agr* system for vesicle lysis.

### Identification of the Toxins Lysing the Lipid Vesicles

**Figure 2:**
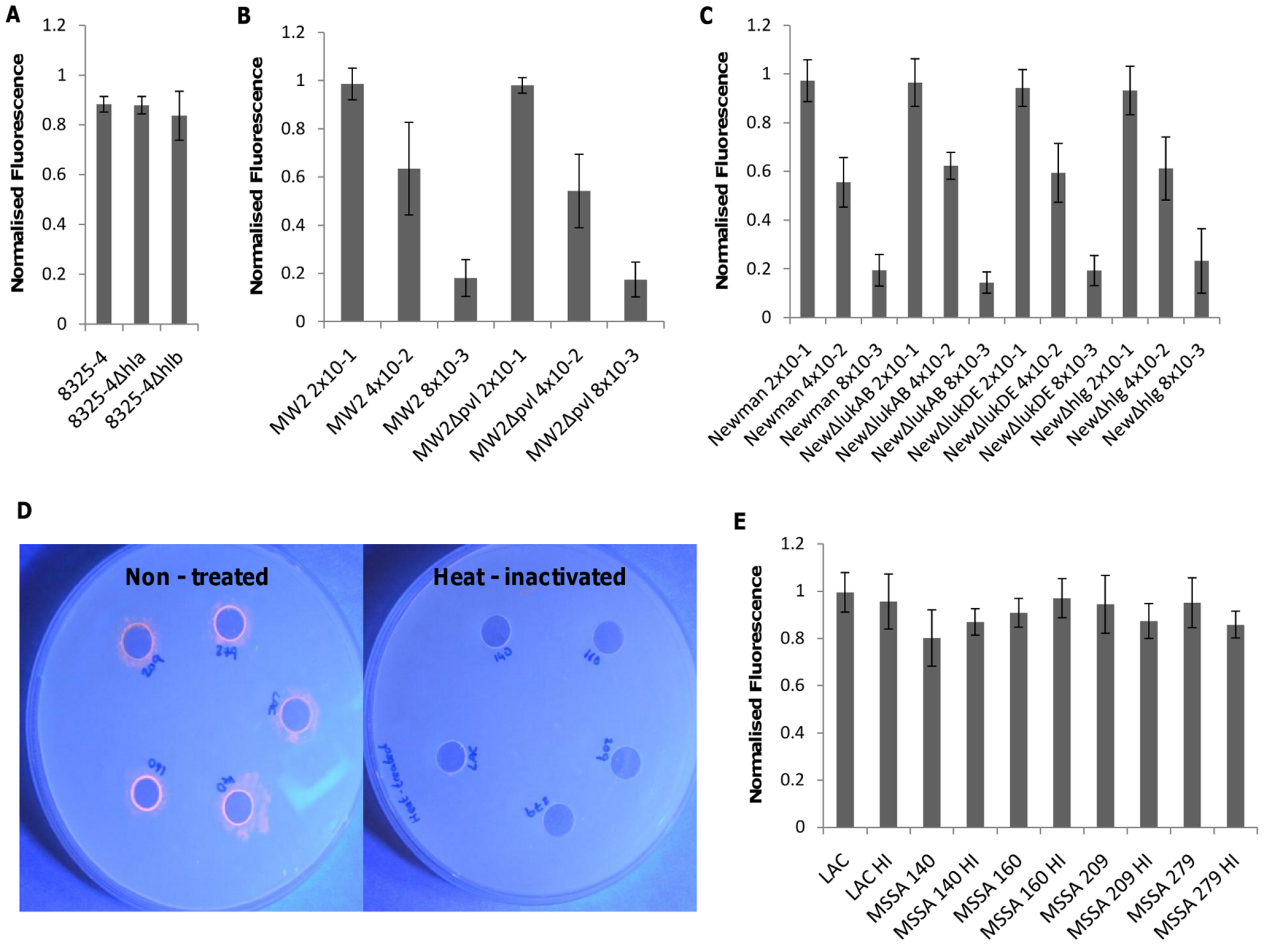
Deducing toxins/enzyme involvement in vesicle lysis. **A)** Deletion of α-or β-toxin had no measurable effect on vesicle lysis. **B)** 5-fold dilutions of MW2 and MW2Δ*pvl* mutant and **C)** Newman, NewmanΔ*lukAB*, NewmanΔ*lukDE* and NewmanΔ*hlgACB* illustrate the lack of involvement of the Panton-Valentine leukocidin and leukocidin AB, leukocidin DE and the gamma-haemolysin in lysis of vesicles. **D)** Phospholipase plate assay showing phospholipase activity as an orange halo around un-treated supernatant filled wells in contrast to no activity with heat-inactivated supernatants. **E)** 95°C Heat-treatment of supernatants retains vesicle lysis ability, suggesting no involvement of either phospholipases or gamma haemolysin in vesicle lysis (see text for details). Lipid vesicles were incubated with bacterial supernatants for 30 min.

*S. aureus* expresses a number of toxins that are regulated by the Agr system. These toxins differ in their mode of action and host receptor specificity, which results in the lysis of different cell types by different toxins. To determine which staphylococcal toxins were causing vesicle lysis, a number of isogenic mutants were assayed (Table 1). The *S. aureus* 8325-4 laboratory strain and the isogenic *hla* (alpha toxin) and *hlb* (beta toxin) mutants were used to assess the role of these two toxins (fig. 2a). It has been known that alpha toxin damages protein free liposomes and that phosphatidylcholine head groups play a vital role in initial association [29], [30]. 1, 2-dipalmitoyl-sn-glycero-3-phosphocholine (DPPC) is a major component of the vesicles under study (53%) and for this reason we explored the role of alpha toxin mediated breakdown of vesicles. Figure 2(a) clearly shows that both the alpha and beta toxin knockout strains caused vesicle lysis at virtually identical levels as the wild type strain, showing that neither toxin were critical in vesicle lysis or caused maximum fluorescence release in these experiments. Alpha toxin mediated lysis requires the clustering of phosphatidylcholine head groups within membrane microdomains enriched in cholesterol and sphingomyelin while the absence of either membrane proteins or the absence of sufficient clustering lead to the inhibition of monomer heptamerization [30] and therefore no lytic event. Furthermore, in the lipid vesicles used here, TCDA was used to stabilize the membrane via cross linking of the acyl chains of DPPC, and this is believed to prevent such head group clustering, rendering the monomers incapable of binding and penetrating the membrane. The beta - toxin is an enzyme which acts as on sphingomyelin [31] and consistent with its activity, no difference in vesicle lysis was observed using the beta - toxin mutant.

**Table 1:** Bacterial strains, peptide sequences and primers used in this study.

Strain/peptide/primer	Description	Reference
***S. aureus***		
RN6390B	NCTC8325 cured of three prophages	[57]
RN6911	RN6390B Δ*agr*::*tetM*	[50]
8325-4	Lab strain NCTC8325 cured of three prophages	[58]
DU1090	8325-4 Δ*hla*::*Em^r^*	[59]
DU5719	β-haemolysin –ve Phage 42E lysogen of 8325-4	[52]
LAC (USA300)	Community acquired MRSA strain	[60]
LAC Δ*hld*	LAC *hld* deletion mutant; *hld* start codon changed from ATG to ATT	[61]
MW2 (USA400)	Community acquired MRSA strain	[60]
MW2 Δ*pvl*	MW2 *ΔlukF/S-PV*::spcm	[60]
MSSA 476	Community-acquired invasive MSSA strain	[54]
RN4220	Restriction negative derivative of 8325-4	[62]
Newman	MSSA lab strain	[63]
NewmanΔ*lukAB*	Constructed using pKOR-1 plasmid	[63]
Newman*ΔlukDE*	Constructed using pKOR-1 plasmid	[63]
NewmanΔ*hlg*	NewmanΔ*hlg*::*tetM*	[64]
***Purified toxins***		
Delta toxin	fMAQDIISTIGDLVKWIIDTVNKFTKK	
PSMα1	fMGIIAGIIKVIKSLIEQFTGK	
PSMα2	fMGIIAGIIKFIKGLIEKFTGK	
PSMα3	fMEFVAKLFKFFKDLLGKFLGNN	
PSMα4	fMAIVGTIIKIIKAIIDIFAK	
PSMβ1	fMEGLFNAIKDTVTAAINNDGAKLGTSIVSIVENGVGLLGKLFGF	
PSMβ2	fMTGLAEAIANTVQAAQQHDSVKLGTSIVDIVANGVGLLGKLFGF	
***Primers***		
*gyr*B Forward	CCAGGTAAATTAGCCGATTGC	
*gyr*B Reverse	AAATCGCCTGCGTTCTAGAG	
RNA III Forward	GAAGGAGTGATTTCAATGGCACAAG	
RNA III Reverse	GAAAGTAATTAATTATTCATCTTATTTTTTAGTGAATTTG	

**Abbreviations:** *agr*, accessory gene regulator; *hla*, gene for α-toxin; *pvl*, gene for Panton-Valentine Leucocidin; *hld*, delta hemolysin; *luk*, leukocidin; *hlg*, gamma haemolysin; PSM, phenol soluble modulin; *gyr*B, DNA gyrase B.

The Panton-Valentine leukocidin (PVL), gamma-haemolysin and the leukocidin family (notably LukAB and lukDE) belong to the bicomponent pore-forming family of toxins. In similar fashion to the alpha-toxin, these leukocidins require specific receptor(s) for lysing biological membranes [32], [33], [34], [35] but may still lyse artificial membranes [36]. Therefore we wanted to investigate whether any measurable difference could be seen between supernatants derived from USA400 MW2 wild type and isogenic PVL mutant and strain Newman and the respective LukAB, LukDE and hlgACB mutants. Due to the high toxin production of these strains and sensitivity of vesicles, supernatants were separated in 5 - fold dilutions. No measurable difference was observed between wild type and mutant strains with our vesicle type. It has been shown previously that gamma-haemolysin can permeate liposomes composed of PC head groups [36], however our results are consistent with two pieces of evidence which also proves that this toxin does not have the capacity to bind and permeabilize this vesicle type. Firstly, the lipid composition is crucial for this toxins’ activity with short (less than 13 carbon atoms) acyl chains being vital for pore-forming ability [37]. The vesicles in question are composed of longer (16 carbon atoms) causing cholesterol to integrate below the DPPC head groups permitting packaging not conducive to monomer binding [37]. Secondly, this toxin, as with alpha-toxin, is susceptible to heat-inactivation at 65°C for 30 minutes [38] and since heat inactivated supernatants were still able to lyse vesicles with the same ability as un - treated supernatant (fig. 2e) over a defined period (20 min exposure), illustrates that the toxin was not involved in lysis.

*S. aureus* also produces a number of enzymes, some with lipolytic activity such as (phospho) lipases. These enzymes are heat-sensitive and to investigate whether these enzymes had an effect on vesicle lysis, we first demonstrated the inactivation of these enzymes in the bacterial supernatant of a selection of clinical isolates following heat treatment using using a lipase plate assay (fig. 2d). These heat-treated supernatants retained their vesicle lytic activities, demonstrating that these enzymes were not the main lytic agent of this system.

**Figure 3:**
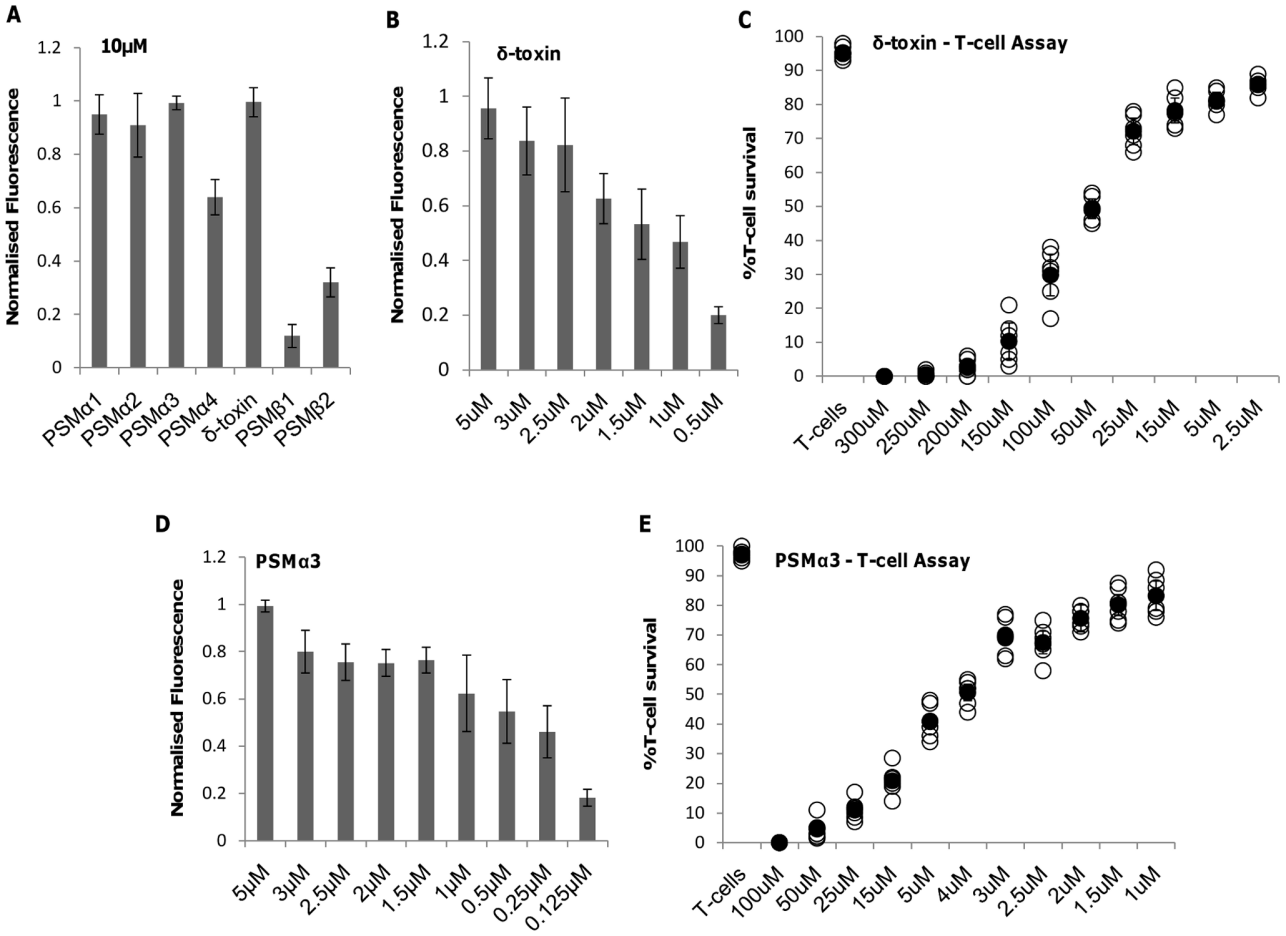
Effect of purified toxins on lipid vesicles and T – cells. **A)** Vesicle rupture as a result of incubation with 10 µM synthetic PSM and delta peptides for 30 min **B)** Lysis of vesicles and **C)** T-cells subjected to selected concentrations of purified delta toxin. **D)** Lysis of vesicles and **E)** T-cells subjected to selected concentrations of purified PSM3α toxin. The vesicle system is highly sensitive to both toxins at low concentrations, while PSM3α is more potent at lysing both vesicles and T-cells than delta toxin. T-cells were incubated for 15 min at 37°C with purified toxins.

This vesicle type was constructed with the aim of being susceptible to delta and PSM toxins and therefore we have eliminated the possible involvement of other known staphylococcal toxins. It has been previously shown that delta toxin can retain its alpha helical structure in phosphatidylcholine bilayers and causes membrane perturbation and lysis in a concentration dependent manner [39]. This was illustrated for delta toxin and also for the PSM alpha toxin 1, 2 and 3 whereas PSMα4 and PSMβ1 and 2 had reduced lytic activity (fig. 3a), which may be due to the structural properties of these peptides and investigations into this is ongoing in our lab. We chose to look closer at delta and PSM3α toxins due to their relevance in immune cell damage, however, PSM alpha 1 and 2 also showed vesicle lysis with similar efficiencies to PSM3α (data not shown). Above a concentration of 2.5 µM and 1.5 µM for delta and PSM3α respectively, vesicle lysis occurs rapidly, whereas below this magnitude, lysis is reduced by approximately 20–30 % of maximum fluorescence (fig. 3b and d). The concentration of delta and PSM3α required to lyse 50 % of vesicles (V_50_) is 1.5 µM and 0.25 µM respectively. By comparing this to the LD_50_ for biologically relevant T - cells which are derived from an immortalized T - cell line [40] the sensitivity of the vesicles to these peptides is illustrated. A 50 - fold (delta) and 16 - fold (PSM3α) increase in toxin concentration is required to cause lysis of 50 % of T - cells (fig. 3(c)(e). It has been shown previously that PSM concentrations of greater than 30 µg/ml were required to cause lysis of human neutrophils [41]. When using PSM3α as an example, this roughly equates to a concentration of approximately 11.5 µM, whereas for the immortalized T-cells, a concentration of approximately 5 µM results in lysis of 50% of T-cells, highlighting the differences in susceptibility of different cell types to PSMs. These differences in susceptibility may be due to the origin of cells, as those experiments using PMN were freshly isolated from human volunteers whereas the T-cells are harvested from *in vitro* growth.

### Comparison of Two Phenotypic Assays in Measuring *agr* Activity

**Figure 4:**
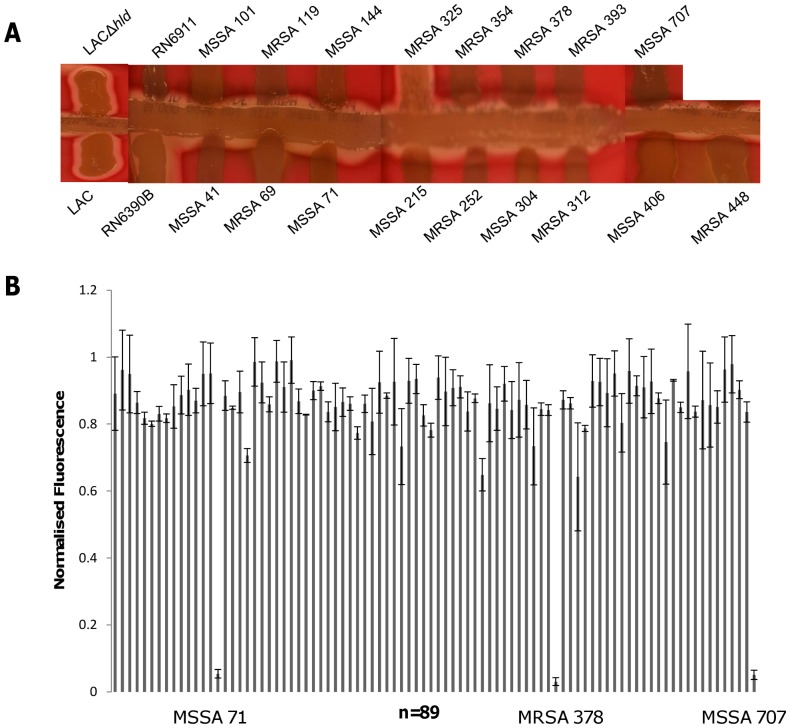
Differences in *agr* activity observed using two methods. **A)** Delta haemolysin plate assay of *agr* positive (RN6390B) and negative (RN6911) strains, 17 *S. aureus* clinical isolates, 16 which are designated as *agr* – negative, one *agr* positive isolate and *agr* – positive LAC and its corresponding isogenic *hld* mutant strain, signifying the effects of delta toxin and PSM on the haemolysin plate assay. **B)** Normalized fluorescence measurements of 89 clinical *S. aureus* strains using the vesicle–supernatant method, highlighting the three strains causing no vesicle lysis.

Having developed an assay that responds to the activity of delta toxin, we wanted to establish how this vesicle lysis test (VLT) assay compared to the conventional delta haemolysin plate CAMP assay for determining Agr function. 89 clinical *S. aureus* strains were tested including 51 hospital acquired and 38 community acquired isolates from diverse genetic backgrounds. Of the 89 strains assayed using the CAMP assay 17.98% (16 of 89) exhibited no synergy between delta and beta haemolysins on the blood agar plates (fig. 4a) and as such were designated as having a dysfunctional Agr system. Using the VLT only 3.3 % (3 of 89) had reduced lytic activity, as measured by fluorescence comparable to the negative control (Hepes buffer) and were classified as *agr* dysfunctional (fig. 4b). Using LAC and its isogenic *hld* mutant it was shown that the synergistic effect of haemolysis could also be observed in the absence of translated delta toxin, an observation that Cheung *et al* have previously shown. These authors have identified *S. aureus* PSMs involvement in the synergistic haemolysis with beta toxin when grown on blood agar [42].

### Validation of the Sensitivity of the VLT by Measuring RNAIII Expression

**Figure 5:**
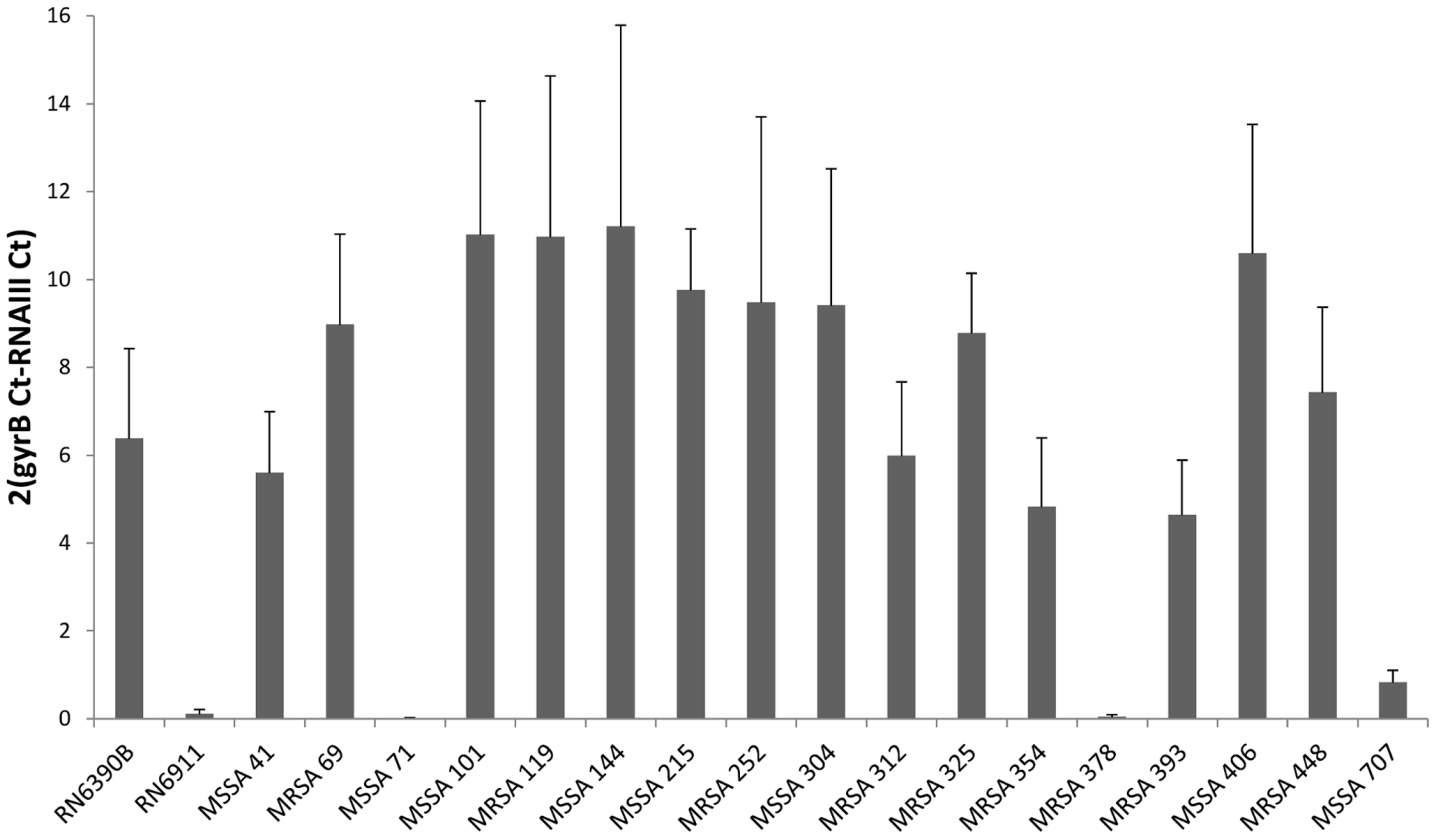
Measurement of *rna*III transcription. Real – time qPCR results of 19 strains consisting of the positive and negative control RN6390B and RN6911 respectively, the 3 negative vesicle lysis test strains (MSSA 71, MRSA 378 and MSSA 707), 13 non-haemolytic strains and the haemolytic strain MRSA 325. Illustrates *rna*III transcription in those strains designated *agr* positive by vesicle method and no transcription is evident in those 3 strains which show no lysis of vesicles.

**Figure 6:**
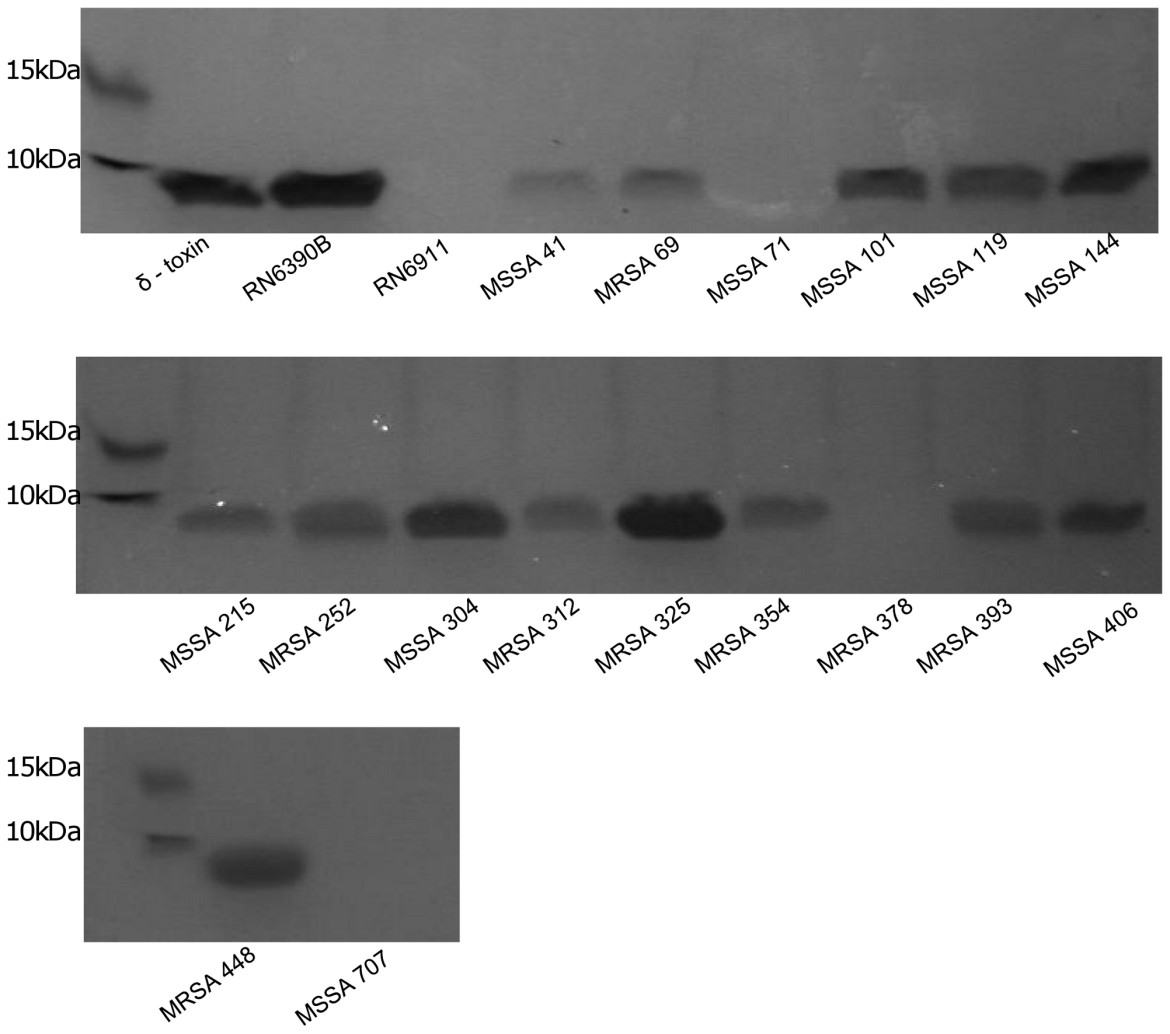
SDS-PAGE of concentrated *agr* regulated peptides. Concentrated and extracted proteins from several *S. aureus* strains, showing the presence of protein bands, indicating a mix of delta and PSM peptides. Purified delta peptide and RN6390B used as a positive control and RN6911 as a negative control. Figure shows the absence of bands in those strains which cause no lysis of vesicles.

To determine which of the two assays (CAMP or VLT) were assaying Agr activity accurately we measured the level of expression on RNAIII in all 16 isolates defined as Agr dysfunctional by the CAMP assay by qRT-PCR (fig. 5). Only the three isolates defined as Agr dysfunctional by both the CAMP and the VLT assays were impaired in the expression of RNAIII (MSSA71, MRSA378 and MSSA707). We also qualitatively assayed the secretion of small amphiphatic peptides (i.e. delta toxin and the PSMs) by these isolates (fig. 6). Although variability existed in the secretion of these peptides across all 16 isolates, only the three designated as being Agr dysfunctional by VLT secreted had no detectable amount of peptide. One of the limiting factors of using this extraction technique is that it may not be sensitive enough to detect all the PSMs, especially if there are low - level expressed. Therefore, we extracted the sample and analysed it by LC/MSMS and identified it as delta toxin. None of the other PSMs were detected in this band.

### *Agr* sequencing

**Figure 7:**
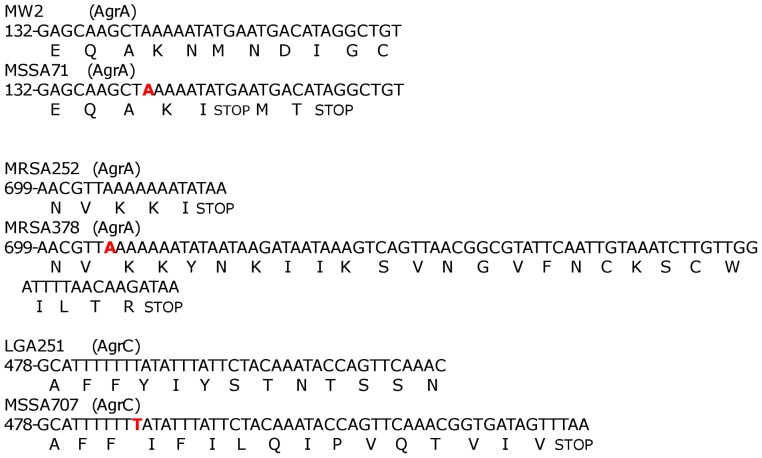
Mutations in VLT negative isolates. Isolates MSSA71, MRSA378 and MSSA707 all contained 1(bold) which led to truncation or alteration of essential agr proteins. The reference strain is given (top) along with the strain in which the mutation was shown to occur in (Bottom). Nucleotide positions are given at the 5’ end of the sequence.

The *agr* locus of all 16 isolates designated as having dysfunctional Agr systesm by the CAMP assay were sequenced. Only the three designated as such by both VLT and CAMP were found to have mutations in this locus (fig. 7). These three strains were from different clonal complexes and therefore we used respective reference strains to align the sequences. All three isolates had 1 bp deletions occurring within a run of adenine (MSSA71 and MRSA378) or thymine (MSSA707) residues. In MSSA71 this lead to a premature stop codon in AgrA truncating the protein at position 49; in MRSA378 this caused frameshift mutation and the addition of 22 amino acids at the C-terminal end of AgrA protein; and in MSSA707 this lead to the truncation of the AgrC protein at position 176. With regards to the other non-haemolytic strains (MSSA41, MRSA69, MSSA101, MRSA119, MSSA144, MSSA215, MRSA252, MSSA304, MRSA312, MRSA354, MRSA393, MSSA406, MRSA448) and haemolytic strain (MRSA325), these had the same *agr* sequence apart from MRSA69 and MRSA325 which were identical and of a different *agr* group ( agr-1 as opposed to agr-3). Therefore differences in *agr* sequence did not account for the differences in haemolytic activity within these two strains.

## Conclusions

Lipid vesicles have also been extensively used to study toxin-membrane interactions [30], [33], [36], [37], [43]. Toxin destruction of biological membranes generally requires specific receptors, a key element in toxin specificity to certain cells. Synthetic lipid vesicles allow different receptor components, such as cholesterol, glycolipids and phospholipids to be incorporated at varying concentrations allowing the possibility of being able to tune the vesicles response to different lytic toxins [44]. In this study we formulate a lipid vesicle that is lysed by a specific group of small, amphipathic, alpha-helical peptides, regulated by the staphylococcal Agr system but unresponsive to other known Agr regulated toxins. This vesicle contains some features consistent with certain eukaryotic membranes, most notably comprising a percentage of cholesterol reflecting that of erythrocyte membranes (20–25% [45]) and featuring two very common membrane phosphoglycerides, phosphatidylcholine and phosphatidylethanolamine. However, our vesicles lack any glycolipids or sphingomyelin, which can be in high proportion in certain cell types [45]. The similarity in composition of our vesicle system to some eukaryotic membranes is reflected in the similar concentrations of purified toxins required to lyse erythrocytes and our vesicles. In general, erythrocytes lysis occurs after incubation with 10 µg/ml (approximately 3.85 µM) of individual PSMs [42], whereas our vesicles require in the range of 1.5–2.5 µM depending on the peptide toxins, both systems requiring incubation at 37°C for 30 min. If we compare this to the results of PSM interaction with POPC vesicles used by Duong *et al* we see a stark difference in the concentration of PSMs used and incubation time required to cause lysis [46]. For POPC vesicles, PSM concentrations were in the range of 0.5 – 1.0 µM and incubated for 200 s to reach maximum lysis. Moreover, the order in which the PSM peptides caused lysis of POPC vesicles is quite different to the order for normal cellular lysis and order of our vesicles, with the PSM β1 and β2 peptides and PSM4α causing the highest degree of lysis in POPC vesicles whereas these peptides have the lowest lytic capabilities under physiological conditions [47] and our vesicles (fig. 3a). The increase in time and toxin concentration required to lyse our vesicle type in comparison to pure POPC is based a number of factors, but largely dependent on the cholesterol composition in our vesicle type. Cholesterol plays a major role in membrane fluidity and the transition temperature of lipids which has an overall impact on the mechanical rigidity of the lipid membrane [48] from which we hypothesise will have a profound effect on toxin - mediate lysis of vesicles and understanding this process is work which is ongoing in our lab. The POPC vesicles are unsaturated and are therefore packed less tightly which may influence toxin binding and disruption of the membrane; unsaturation also lowers the transition temperature and is more prone to oxidation [49] again affecting membrane integrity.

Agr dysfunction is an increasingly important issue due to its relevance with regard to persistent bacteraemia and decreased antibiotic glycopeptide susceptibility such as to vancomycin [19], [20]. Loss of Agr activity is also implicated in increased biofilm formation and attachment to inert surfaces, which is important as the majority of bloodstream hospital acquired infections are catheter associated [50], [51]. However, the majority of these studies looking at correlations between Agr dysfunction and clinical outcomes use the CAMP assay.

We suggest that the use of the CAMP assay for determining Agr activity may not be sufficiently sensitive in determining Agr function. Using our VLT assay we correctly identified the only 3 isolates from a collection of 89 that had mutations in the Agr locus (summarised in Table 2). A possible confounder of the VLT method is their sensitivity to the other PSM toxins. It has been shown previously that *psm* genes can be regulated in an RNAIII-independent manner, with AgrA being important in this up-regulation [52]. However, this also affects the outcome of the CAMP assay, as we show in Fig. 4a. We must also keep in mind that the vast majority of strains that produce detectable levels of PSMs are the highly toxic community acquired type IV SCCmec strains, which produce considerably higher concentrations of delta toxin than PSM [47]. No examples of Agr dysfunction have been reported amongst this group, indeed Agr function is believed to be critical to their emergence and virulence [53], suggesting that neither the VLT or the CAMP assay will be of use clinically for these types of infection.

**Table 2:** Key characteristics of CAMP and VLT assayed strains.

Strain	CC	Agr type	CAMP	VLT (N Fluor)¶	RNAIII Expression*	Agr locus/ mutation
MSSA41	30	III	Negative	0.71±.04	5.7±1.4	Identical to MRSA252
MRSA69	30	I	Negative	0.95±.09	8.9±2.1	Identical to MRSA325
**MSSA71**	**1**	**III**	**Negative**	**0.05±.01**	**0.02±.01**	**-1bp(nt147) AgrA**
MSSA101	30	III	Negative	0.98±.07	11±3.1	Identical to MRSA252
MRSA119	30	III	Negative	0.85±.02	10.9±3.7	Identical to MRSA252
MSSA144	30	III	Negative	0.99±.07	11.2±4.6	Identical to MRSA252
MSSA215	30	III	Negative	0.80±.09	9.7±1.4	Identical to MRSA252
MRSA252	30	III	Negative	0.73±.11	9.5±4.2	MRSA252
MSSA304	30	III	Negative	0.91±.03	9.4±3.1	Identical to MRSA252
MRSA312	30	III	Negative	0.65±.05	5.9±1.7	Identical to MRSA252
MRSA325	30	I	Positive	0.92±.05	8.8±1.4	Identical to MRSA69
MRSA354	30	III	Negative	0.73±.11	4.8±1.6	Identical to MRSA252
**MRSA378**	**30**	**III**	**Negative**	**0.03±.01**	**0.05±0.03**	**-1bp(nt705) AgrA**
MRSA393	30	III	Negative	0.64±.16	4.64±1.2	Identical to MRSA252
MSSA406	30	III	Negative	0.92±.07	10.6±2.9	Identical to MRSA252
MRSA448	30	III	Negative	0.95±.14	7.4±1.9	Identical to MRSA252
**MSSA707**	**16**	**II**	**Negative**	**0.05±.02**	**0.83±.3**	**-1bp(nt487) AgrC**

Abbreviations: CC; Clonal complex, CAMP; synergistic haemolysis plate assay, VLT; vesicle lysis test.

¶ Values shown in normalised fluorescence units.

* qRT-PCR after 8 hours.

The question that this study also touches on is how much Agr activity is required to determine a strain to be functional, semi-functional or have complete inactivity? We have suggested that those strains which were VLT negative and non-haemolytic were the only true Agr inactive isolates, acquiring mutations leading to truncation of essential proteins. This VLT is a sensitive, rapid assay which is quick and easy to perform and amenable to 96-well plate high-throughput analysis and may help us in redefining the Agr activity of clinical isolates.

## Materials and Methods

### Bacterial Strains and Culture Conditions

Frequently used bacterial strains are listed (Table 1). Wildtype *S. aureus* strains isolated from patients with invasive disease (51 hospital acquired and 38 community acquired) within Oxfordshire, United Kingdom, between 1997 and 1998 are described previously [54]. All strains were routinely stored at −80°C in 15 % glycerol/broth stocks until required. *S. aureus* strains were streaked onto Tryptic Soy agar and single colonies transferred to 5 mL Tryptic Soy broth and propagated in a shaking incubator for 18 h at 37°C. Bacterial growth was measured at OD_600__nm_ at 37°C and shaking at 300 rpm in Costar 96-well round – bottomed plates in tandem with fluorescence using a dual absorbance/ fluorescence script. To identify specific roles of toxins in vesicle lysis, bacterial supernatants were heated to 95°C for one hour.

### Vesicle Formulation

Vesicle suspensions were prepared by mixing lipid and fatty acid components in chloroform: 25 mol % of 10, 12-Tricosadiynoic acid, 53 mol % 1, 2-dipalmitoyl-sn-glycero-3-phosphocholine, 2 mol % 1, 2-dipalmitoyl-sn-glycero-3-phosphoethanolamine and 20 mol % of cholesterol. The dried lipid was rehydrated using 10 mL of 50 mM 5(6)-carboxyfluorescein (CF) in HEPES buffer solution, vortexed and heated in a hot water bath at 75°C for 10 minutes. Three freeze/thaw cycles were carried out by initially immersing the lipid-containing vial in liquid nitrogen and then heating the vial back to room temperature to homogenize the solution [55]. A turbid solution was an indication of vesicle formation. Vesicles were then extruded 3 times at 50°C using a Liposofast vesicle extruder through 2 × 0.1 µm polycarbonate filters under nitrogen pressure. The initial opaque vesicle solution becomes translucent after extrusion. The vesicle solution was then purified using illustra Nap-25 columns (GE Healthcare) to remove unencapsulated CF dye. The columns were washed with 15 mL of HEPES buffer to remove the 0.15% Kathon CG/ICP biocide. 2 mL of vesicle solution was added to each column and allowed to load. 1 mL of HEPES buffer was then added and left to drain. After this a further 2 mL of HEPES buffer was added and the resultant pure vesicle solution was collected and stored at 4°C overnight. These 10, 12-tricosadiynoic acid vesicles were then cross-linked using a CL1000 Ultraviolet crosslinker on setting 1 for 6 seconds. All chemicals were purchased from Sigma-Aldrich and lipids from Avanti Polar lipids. As a quick method to illustrate CF encapsulated vesicles, 0.01% v/v Triton X-100 and HEPES was used as a positive and negative control respectively.

### Vesicle Toxicity Assay

The vesicle toxicity assay was designed *de novo* were experimental conditions were optimised before use. Fluorescence intensity was measured at excitation and emission wavelengths of 485–520 nm respectively on a FLUOROstar fluorimeter (BMG Labtech). Depending upon the experiment, two different assays were employed, one utilising whole bacterial cells and the other using bacterial culture supernatant. In the first method, bacterial culture was normalised to a specific colony forming unit/ml (cfu/ml) achieved through correlation with optical density. An overnight culture (18 h) washed twice in PBS and diluted to an OD of 0.1 which represented 10^6^ CFU/ml. This was serially diluted to 10^5^ and 10^4^ CFU/ml in TSB. 200 µl of bacterial culture was added to 50 µl of vesicle solution in triplicate. The fluorescence of each sample was then measured at 5 min intervals for 18 h. Positive and negative controls were pure vesicles with 0.01% Triton X-100 and HEPES respectively. For the supernatant assay, bacteria were grown for 18 h, supernatant was harvested by centrifugation at 14000 rpm for 10 min and filter sterilized through a 0.22 µm filter. 50 µl of vesicle solution was incubated with 50 µl of bacterial supernatant or differing concentrations of purified delta toxin or PSMs (Biomatik; 99% purity) and measured for 30 min with the above parameters. Normalised fluorescence was achieved using the equation (Ft–F0)/(Fm/F0) where Ft is the average fluorescence value at a specific time point, F0 is the minimum and Fm is the maximum fluorescence value in that particular experiment.

### Lipase Plate Assay

Lipase plate assay was designed as described previously with some minor modifications [56]. Olive oil (1 %) and rhodamine B (0.001 %) were used as substrate and added to the agar medium after sterilization and cooling to 60°C. 3-mm-diameter holes were punched into the agar and 50 µl of cell-free supernatant harvested from 18 h stationary phase bacterial cultures were added and left to incubate for 18 h at 37°C. Plates were irradiated with a UV light and images captured on a Nikon camera.

### CAMP Assay

The conventional method to determine or measure *agr* functionality is via delta – haemolysin production. This was determined by streaking the beta – haemolysin positive *S. aureus* RN4220 strain on washed sheep blood agar (SBA) plates. The test strains are streaked perpendicular to this strain and any enhanced zone of haemolysis where the delta lysin [22] or PSM peptides[Cheung] overlaps with the beta – haemolysin zone was scored positive for delta haemolysin production and thus *agr* activity [22].

### T-cell Toxicity Assay

To analyse the difference in sensitivity between a biological membrane and vesicles to purified toxins, an adaptation of the T-cell toxicity assay was used as described previously [40]. All experiments were done in duplicate with three biological repeats.

### RNA Isolation

Cultures of *S. aureus* grown overnight were diluted 1∶1000 into fresh TSB and grown at 37°C for 8 h (late exponential phase) at which time samples were collected for RNA isolation. Cultures were treated with two volumes of RNAprotect (Qiagen) incubated at room temperature for 10 min, centrifuged and resuspended in Tris 0.05 M (pH 7.5) and the pellet further treated with 300 µl of EDTA 0.5 M/ lysostaphin (5 mg ml^−1^) and incubated for 1 h. RNA was then isolated using the Qiagen RNeasy Midi Kit according to the manufacturer’s instructions with the addition of Turbo DNase (Invitrogen) after the purification step. The RNA was quantified using the Qubit RNA assay kit (Invitrogen) and the absence of DNA verified by PCR using standard primers.

### Reverse Transcription and qRT-PCR

Reverse transcription of messenger RNA to complementary DNA (cDNA) was generated using the SuperScript II Reverse Transcriptase (Invitrogen) according to manufacturer’s instructions using random hexamers (Qiagen). Primers used for gyrase B (gyrB) and RNA III are listed in Table 1. Standard curves were generated for both primer sets on serial dilutions of cDNA to determine primer efficiency. The reverse-transcriptase PCR (RT-PCR) was performed as follows: 5 µl cDNA, 7.5 µl SYBR reagent (BioRad), 0.5 µl forward and reverse primer and RNase-free water (Sigma) to a total volume of 15 µl. The cDNA was subjected to real-time PCR using the Applied Biosystems Step-One Real Time PCR detection system (Applied Biosystems). Cycling conditions were 95°C for 10 min followed by 40 cycles of 95°C for 15 s and 60°C for 1 min and a dissociation step 95°C for 15 s and 60°C for 1 min. Cycle threshold values were determined for 3 biological repeats in duplicate. For each reaction, the ratio of RNA III and gyrB transcript number was calculated as follows: 2^(Ct gyrB – Ct RNAIII)^.

### Peptide Extraction and SDS-PAGE

Overnight cultures of *S. aureus* were diluted 1∶1000 into 50 ml TSB and grown at 37°C for 18 h, after which cultures were centrifuged and 30 ml of cell – free supernatants were mixed with 10 ml 1-butanol. Extraction was performed by shaking mixed solutions for 3 h at 37°C. Samples were then centrifuged for 3 min for complete separation and the upper organic phase was collected, aliquoted into 1 ml tubes and concentrated using a vacuum oven overnight at 40°C. Dried samples were dissolved in 200 µl of 8 M urea. Aliquots were mixed with 2×Laemmlli sample buffer and heated at 95°C for 5 min before being subjected to SDS-PAGE (10% acrylamide). This was performed in triplicate and a representative coomassie stained gel can be seen in fig. 6. Bands were excised and sent for LCMSMS at the Central Proteomics Facility at the University of Oxford.
